# Progress towards measles and rubella elimination in Lesotho, 2011–2025

**DOI:** 10.1371/journal.pone.0358555

**Published:** 2026-09-16

**Authors:** Yankuba Singhateh, Tshepiso Mechele Matoko, Mannini Shaabe, Selloane Amelia Maepe, Francis Dumobong Ngmenasong Abobo, Sarah Waithera Wanyoike

**Affiliations:** 1 Vaccine Preventable Disease Program, East and Southern Africa Inter-Country Support Team, World Health Organization, Harare, Zimbabwe; 2 Epidemiology and Disease Control Unit, Directorate of Public Health Services, Ministry of Health, Banjul, The Gambia; 3 National Immunisation Program, Ministry of Health, Maseru, Lesotho; 4 World Health Organization Lesotho Country Office, Maseru, Lesotho; 5 Independent Consultant, Maseru, Lesotho; Regional Health Care and Social Agency of Lodi, ITALY

## Abstract

**Background:**

Lesotho, a lower middle-income country in southern Africa, has historically had relatively high routine immunization coverage over the last three decades. Since 1996, the country has been implementing measles elimination strategies. This manuscript evaluates Lesotho’s performance, from 2011 to 2025, against the World Health Organization’s criteria for measles and rubella elimination.

**Methods:**

We conducted a retrospective review of Lesotho’s immunization and surveillance data during 2011–2025. This included Lesotho’s annual WHO/UNICEF Estimates of National Immunization Coverage (WUENIC) for routine measles-containing vaccine (MCV) doses; performance in the periodic measles rubella vaccination campaigns and case-based surveillance; and the trends of measles and rubella incidences.

**Results:**

During 2011–2025, MCV1 coverage generally remained ≥90% but failed to reach the 95% elimination target. MCV2 coverage reached 80% in 2011 and 2012 before declining in subsequent years. Lesotho has mostly attained the targets for surveillance performance according to the two principal performance indicators – non-measles febrile rash illness rate and the proportion of districts that report a minimum of one suspected case. From 2011 to 2015, measles incidence stayed below one per 1 million population in Lesotho. However rubella incidence ranged between 33.4 in 2012 to 142.3 in 2014, and declined significantly after MR introduction in 2017.

**Conclusion:**

Lesotho has made significant progress in measles and rubella elimination efforts. To sustain the gains made, the country should work to strengthen vaccination in the second year of life, reinforce demand generation during preventive campaigns, address population denominator inaccuracies that hinder accurate coverage tracking, and systematically document measles and rubella virus genotypes.

## 1. Introduction

Lesotho is a mountainous landlocked country with an estimated total population of 2,379,237 people as of 2026 [1]. It is a lower-middle-income country with a GDP per capita of 1,001 USD by 2024 [2]. Lesotho has one of the world’s highest HIV infection rates with a 25.6% adult prevalence as of 2022 [3]. The country is divided into 10 districts each managed by a district health management team that oversees implementation of public health activities including immunization.

Immunization is one of the pillars of Lesotho’s national health policy and a primary strategy for improving child survival. Lesotho has an estimated 42,540 annual live births, as of 2025 [1]. Since the launch of the Expanded Program on Immunization (EPI), immunization services have been provided free-of-charge. Of the 315 total functional health facilities, 214 were providing immunization services as of 2023 [2]. The health facility-based service delivery is the predominant service delivery strategy for routine immunization. But non-facility-based strategies (including outreach and mobile sites) remain essential for reaching remote and hard-to-reach communities [2,4].

The first dose of measles-containing vaccine (MCV1) has been part of the EPI schedule since its inception and is administered at nine months of age. The second MCV dose (MCV2), given at 18 months, was introduced in 2001. Rubella-containing vaccine (RCV) was introduced in 2017, leading to the replacement of the monovalent MCV with the combined measles-rubella (MR) vaccine, as recommended by WHO [4].

The African Regional goal of measles elimination, is defined by the programmatic targets of achieving a minimum of 95% coverage of 2 timely administered MCV doses, attaining the performance standards for measles case based surveillance, and reducing measles incidence to less than 1 case per million population. This goal has been included and endorsed in the global Immunization Agenda 2030 (IA2030) [5,6]. The commitment towards measles elimination has been made by all 6 WHO regions and over 60 million measles-related deaths have been prevented by immunization worldwide between 2000 and 2025 [6].

Despite global progress in reducing measles cases and deaths, member countries of the WHO African Region have had challenges in meeting the regional measles-rubella elimination targets [5,6]. A significant milestone was achieved in 2025 when Cabo Verde, Seychelles and Mauritius became the first sub-Saharan African countries to join the 94 and 133 other countries around the world to have attained measles and rubella elimination statuses, respectively. This means that the 3 countries, as documented by a standard functional case-based surveillance system, were able to confirm they have interrupted endemic measles and rubella transmission for at least 36 months [7].

The verification of the three countries comes in the background of Regional progress registered in measles elimination over the years, with an estimated 91% reduction in measles-related deaths in the African Region between the years 2000 and 2024 [6]. Countries in the southern African sub-region have historically had relatively high routine immunization coverage and lower measles incidences compared to the rest of the African Region. In 2002, following the initial few years of implementation of measles mortality reduction strategies in southern Africa, including in Lesotho, it was reported that measles incidence declined to less than one per million population [8]. However, in the past two decades, recurrent measles outbreaks have been documented in countries in the southern African subregion [9]. This manuscript examines Lesotho’s performance through the end of 2025, against the World Health Organization’s criteria for measles elimination.

## 2. Methods

### Routine immunization

The study utilized a retrospective design to evaluate Lesotho’s immunization landscape. Routine immunization data were analyzed using the WHO and UNICEF Estimates of National Immunization Coverage (WUENIC) for MCV1 and MCV2 from 2011 to 2025. These estimates are derived from administrative data aggregated from health facility records to the national level and supplemented by available survey results [10,11].

### Supplemental immunization

Due to the sub-optimal routine immunization coverage, there is continuous accumulation of measles susceptible children. Supplemental immunization activities (SIAs) are therefore implemented periodically; every 2–4 years using different strategies to reach every eligible child. During SIAs, vaccination teams do tally each vaccine dose given and at the end of each vaccination day, aggregate data is transmitted to the next higher levels (district and national levels). This study reviewed and analysed the MCV administrative coverage and post-campaign coverage survey (PCCS) estimates of SIAs conducted in Lesotho during 2011–2025.

### Measles surveillance and disease incidence

Measles and rubella case-based surveillance data were extracted from the WHO AFRO database for the 2011–2025 period. Vaccine preventable diseases surveillance, including measles case-based surveillance, is under the purview of the National Immunisation Program of Lesotho. Measles case-based surveillance reports originate at and are passed from the health facility level to the respective upper levels until it reaches the national level. Samples collected from suspected cases are processed at the national measles serology lab. The Measles Serology Laboratory of Lesotho is part of the network of laboratories in the WHO African Region using a standardized testing protocol [12]. According to the protocol, samples taken from suspected measles cases who meet the standard case definition should first be tested for measles-specific immunoglobulin M (IgM) antibodies. Sample specimens that test negative for measles are tested for rubella IgM. In addition to measles IgM positivity, measles cases are confirmed by epidemiological linkage and based on clinical compatibility, as per the WHO African regional measles surveillance guidelines [13].

Surveillance sensitivity was measured against two principal performance indicators. The non-measles febrile rash illness rate which has a target of at least 2 cases per 100,000 population; and the proportion of districts reporting at least one case of suspected measles with a blood specimen, with a target of at least 80% per year [13]. The impact of measles and rubella elimination is monitored by calculating the annual incidence rate of confirmed measles and confirmed rubella, expressed as rates per 1 million population.

## 3. Results

### Immunization activities

**Fig 1:**
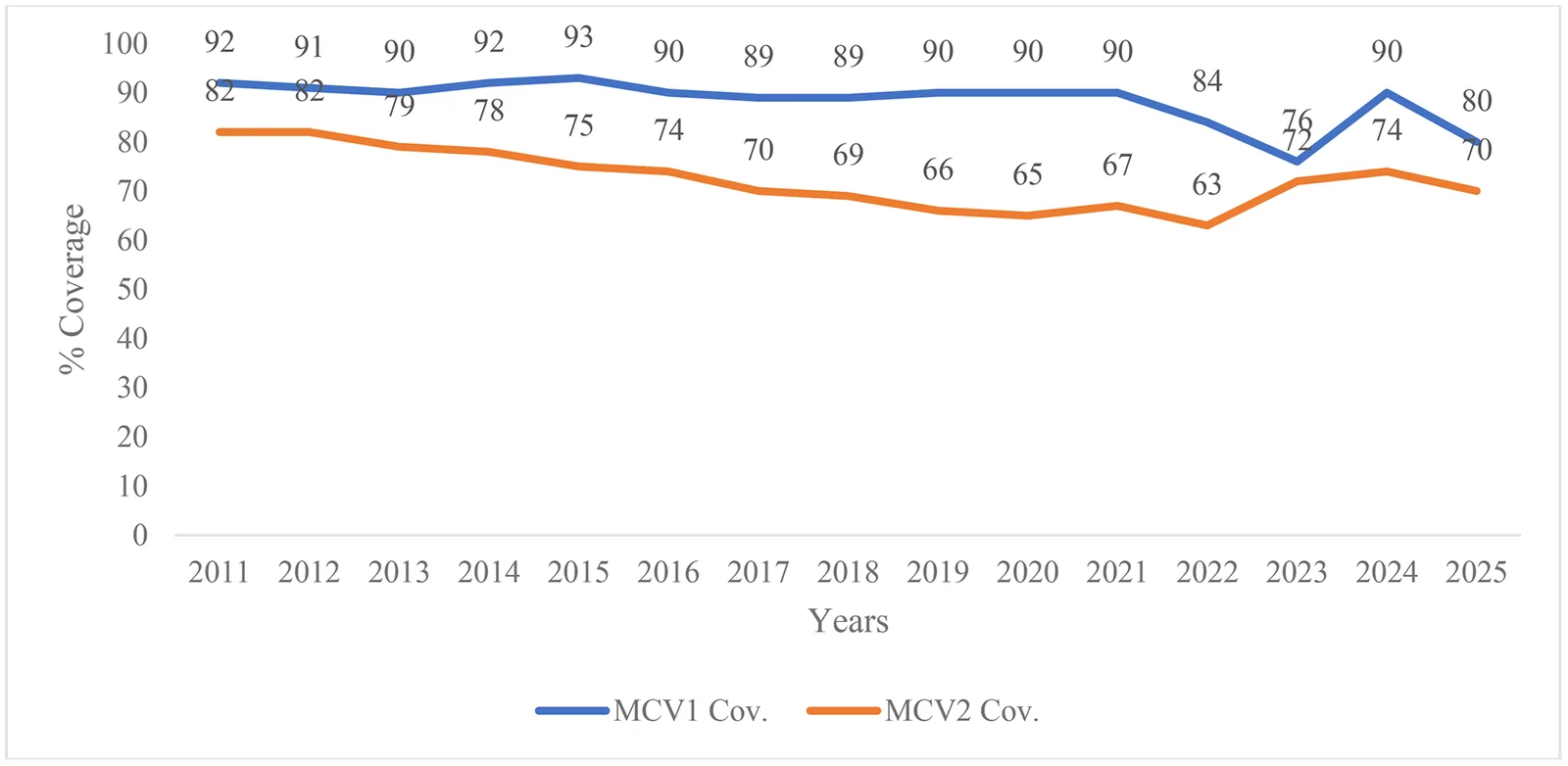
WHO UNICEF Coverage Estimates for First and Second Dose Measles Vaccination, Lesotho, 2011–2024.

From 2011 to 2016, first dose of measles-containing vaccine (MCV1) coverage consistently reached ≥90% in Lesotho but fell short of the 95% coverage target. The lowest MCV1 performance levels, for the period under review, were recorded in 2022 and 2023 at 84% and 76% respectively. The country’s MCV1 coverage recovered back to 90% in 2024. During 2011–2024, MCV2 coverage of ≥ 80% was registered in 2011 and 2012 with MCV1-MCV2 dropouts of 10% and 9% respectively. Thereafter, MCV2 coverage was consistently lower than 80% with high MCV1-MCV2 dropout rates (>10%) during 2013–2024 except for 2023 (Fig 1).

Since the initial phased measles catch-up campaign in 1999–2000, during which 628,938 children aged 9 months to 14 years were vaccinated, nationwide supplemental immunization activities (SIAs) have been conducted in Lesotho at intervals of 2–5 years [4]. These periodic campaigns have helped sustain population immunity over time. Between 2010 and 2025, all but one of the SIAs conducted were follow-up campaigns mostly targeting children 9–59 months of age. In 2017, Lesotho implemented the measles-rubella catch-up campaign which targeted children 9–14 years. Administrative coverage estimates for these measles and rubella campaigns ranged between 45.1% in 2022 to 91.0% in 2010. No post campaign coverage survey (PCCS) was conducted to validate the administrative coverage estimate for the 2022 campaign. The PCCS results for the remaining 4 SIAs ranged from 83.7% in 2025 and 94.3% in 2010 (Table 1).

**Table 1:** Administrative and survey coverage results from previous measles vaccination campaigns, lesotho, 2010-2025.

Year	Activity Type	Target Age Group	Scale of SIAs	Number of children vaccinated	% Reached (Admin Cov.)	% Districts with ≥95% coverage	Survey results (%)
2010	Follow-up	6 months -14 years	Nationwide	558,335	91.0	40.0	94.3
2013	Follow-up	9–59 months	Nationwide	147,676	72.3	90.0	92.0
2017	Catch-up	9 months -14 years	Nationwide	540,017	79.6	30.0	92.0
2022	Follow-up	9–59 months	Nationwide	85,582	45.1	0.0	
2025	Follow-up	9–59 months	Nationwide	117,796	68.0	10.0	83.7

### Surveillance activities

The measles-rubella case-based surveillance system in Lesotho has been functional for more than two decades. The national non-measles febrile rash illness rate has consistently been above the minimum threshold of 2 per 100,000 population. During 2011–2025, Lesotho’s non-measles febrile rash illness performance ranged from 2.5 in 2016 to 36.9 in 2023 (Table 2). Over the same period, the proportion of districts that reported a minimum of 1 suspected measles case with a blood specimen was consistently ≥ 80% with the exception of 2011 and 2021 when the 80% target was not met (Table 2). Lesotho has yet to establish congenital rubella syndrome (CRS) sentinel surveillance or conduct a retrospective review of records to establish the burden of CRS disease in the country [2].

**Table 2:** Non-Measles Febrile Rash Illness Rate and Proportion of Districts Reporting by Year, Lesotho, 2011–2025.

Year	Target population	Suspected Measles cases	Confirmed Measles cases	Non-Measles Febrile Rash Illness Rate (Target: ≥2/100,000 Pop.)	% districts reporting at least one suspected case of measles with blood specimen (Target: ≥80%)
2011	1,806,833	169	0	9.4	70%
2012	2,034,355	179	0	8.8	90%
2013	2,056,504	503	1	24.4	100%
2014	2,080,089	523	0	25.1	100%
2015	2,104,612	273	1	12.9	90%
2016	2,130,422	77	13	3.0	90%
2017	2,157,111	77	1	3.5	80%
2018	2,183,603	135	1	6.1	100%
2019	2,209,406	299	6	13.3	91%
2020	2,235,727	107	0	4.8	100%
2021	2,261,542	57	0	2.5	64%
2022	2,286,111	262	3	11.3	91%
2023	2,311,472	868	15	36.9	91%
2024	2,337,423	413	1	17.6	91%
2025	2,363,325	414	23	16.5	91%

### Reported measles and rubella incidence

From 2011 to 2015, Lesotho’s measles incidence remained below one per 1 million population. During the same period, almost all suspected measles cases tested negative for measles but a significant number were IgM positive for rubella (incidence ranged between 33.4 in 2012 to 142.3 in 2014). During 2016–2019, Lesotho’s measles incidence remained below 5 per 1 million population. However, there was a significant increase in incidence immediately after the COVID-19 pandemic, with 2023 and 2025 recording 7.4 and 9.7 measles incidences respectively [4]. Rubella incidence declined from 9.9 in 2016 to less than 1 in 2018 following the introduction of the rubella-containing vaccine in 2017 [4]. However, rubella incidence showed an increase in 2022 and 2023. (Table 3).

**Table 3:** Epi-classification of cases and incidence/1,000,000 population of measles and rubella, Lesotho, 2011–2025.

Year	Target population	Suspected Measles cases	Confirmed Measles cases	Confirmed Rubella cases	Incidence of measles/ million	Incidence of rubella/ million
2011	1,806,833	169	0	78	0.0	43.2
2012	2,034,355	179	0	68	0.0	33.4
2013	2,056,504	503	1	166	0.5	80.7
2014	2,080,089	523	0	294	0.0	141.3
2015	2,104,612	273	1	129	0.5	61.3
2016	2,130,422	77	13	11	6.1	5.2
2017	2,157,111	77	1	8	0.5	3.7
2018	2,183,603	135	1	0	0.5	0.0
2019	2,209,406	299	6	4	2.7	1.8
2020	2,235,727	107	0	0	0.0	0.0
2021	2,261,542	57	0	0	0.0	0.0
2022	2,286,111	262	3	9	1.3	3.9
2023	2,311,472	868	15	9	6.5	3.9
2024	2,337,423	413	1	2	0.4	0.9
2025	2,363,325	414	23	1	9.7	0.4

## 4. Discussion

This study evaluates Lesotho’s performance with regards to the strategies for measles and rubella elimination against WHO Africa Region’s elimination targets. These targets include attaining a minimum of 95% MCV coverage in routine immunization and in all slated measles SIAs including outbreak response immunizations; registering measles incidence below 1 per 1,000,000 population; achieving a minimum non-measles discard rate of 2 per 100,000 population; and ensuring that at least 80% of its districts investigate a minimum of 1 suspected measles case per year [6,7].

During most of the years of the study period (2011–2025), Lesotho had maintained routine immunization coverage estimates of 90% and above for MCV1. However, the same level of performance was not recorded for MCV2 [10]. Lesotho will need to devise strategies to improve and sustain high routine immunization coverage with both doses of MR vaccine as part of the elimination efforts [13]. Currently, Lesotho provides the 4^th^ dose of Diphtheria-Tetanus vaccine (DT4 booster dose) and vitamin A supplementation in the second year of life alongside MCV2 [14]. Studies have found that countries offering more antigens and interventions in the second year of life were more likely to improve MCV2 coverage and as such bridge the gap between MCV1 and MCV2 performance [15]. Thus, implementing a structured second year of life platform for the provision of child survival interventions, alongside regular programmatic supervision, and introducing additional high priority interventions in the well-child visit at 18 months of age could help boost MCV2 coverage and reduce drop-out rates.

The HIV burden in Lesotho is one of the highest in the world with considerably higher prevalence recorded among women of childbearing age (between 15 and 49 years old) than their male counterparts [3]. Studies have shown that maternal antibodies against measles wane faster among HIV-infected women making infants born to them more susceptible to measles infection at an early age [16]. This has implications to the immunity profile of young infants in countries like Lesotho. The WHO measles vaccine position paper recommends providing a first supplementary dose of measles vaccine (MCV0) as early as at 6 months of age in areas with a high incidence of both HIV infection and measles, to be followed by the 2 routine doses of MCV (MCV1 and MCV2) according to the national immunization schedule [12].

Survey coverage estimates have always been higher than the reported administrative coverage for both routine and supplemental immunization activities in Lesotho, which is suggestive of the existence of denominator inaccuracies. Similar trends were observed in Eritrea with survey results consistently higher than administrative coverage estimates due to inaccurate denominators [17]. It is therefore imperative that the denominator issue is addressed, either through a nationwide census or through scientifically sound methods, in order to obtain credible coverage estimates for both routine and supplemental immunization activities.

The most recent increase in measles cases that occurred in 2023 was attributable to the long interval between SIAs – 2017 to 2022 – resulting in the critical accumulation of unvaccinated measles susceptible children [4,12,13,18] and the suboptimal performance in the 2022 SIAs which had 46% coverage. The 2022 SIAs was inadequately planned and resourced, and also sub-optimally implemented owing largely to the rush and the urgency to integrate the MR campaign with COVID-19 vaccinations without implementing robust social mobilisation and communication strategies as part of the preparation to the campaign [19]. The implication of this low coverage after a long interval without a campaign is the persistence of immunity gaps among children including school age groups.

Moreover, the country has faced recurrent risks of measles outbreaks given that neighbouring South Africa has been having disruptive measles outbreaks, affecting several provinces in the country since 2022, as well as a large-scale rubella outbreak that occurred in 2024 [20]. Countries in the southern African sub-region have had concurrent multi-country measles outbreaks in the past, owing to cross border population movements and the existence of vaccine-hesitant groups in some countries [8,21]. The large measles outbreak of 2009–2010 had a very clear progression between countries in the subregion, with the eventual involvement of 7 countries [8]. Recurrent re-introduction and importation of measles virus has been documented in countries that had even attained elimination status in other WHO Regions [22–24]. It is therefore essential that a synchronized regional approach to measles-rubella elimination strategy be adopted and implemented by all southern African member countries.

The 2025 MR campaign - with a PCCS result of 84% - fell short of the 95% coverage target. Factors cited to have been responsible for this included information gaps to caregivers on the eligibility for vaccination [25]. Thus, there is a need for strong emphasis on timely and proper social mobilisation and messaging in the run up to future campaigns so as to ensure that eligible children are not missed.

Both major surveillance sensitivity measures reviewed in this study – the non-measles, non-rubella discard rate and the proportion of districts that report a minimum of 1 suspected case – have been met for most of the years under review. This underscores that the measles case-based surveillance system in Lesotho is quite sensitive. With the current levels of low levels of transmission of measles and rubella, surveillance officers should strive to thoroughly investigate each and every suspected measles case. To date, Lesotho does not yet have any documented measles or rubella virus genotype strains in the global databases. The country should invest in collecting the appropriate specimens for molecular testing in the appropriate Regional Measles and Rubella Reference Laboratory. This will be critical to determine the specific source of any cases in the future, and for the verification of endemic disease transmission.

WHO’s established framework for the verification and documentation of progress towards measles and rubella elimination indicates that countries should establish an independent national verification committee (NVC) to continuously track the implementation of strategies and oversee the documentation of progress [26,27]. It is therefore imperative that Lesotho establishes an NVC to start documenting the progress towards measles and rubella elimination and utilize the process to reinforce political will and technical oversight to strengthen the implementation of the elimination strategies.

### Limitations

This study has some limitations. First, administrative coverage data may not have accurately reflected actual performance due to the inaccuracies in the denominators as shown by the PCCS results. This is most problematic with campaign coverage results that were not validated with post-campaign coverage surveys. Second, this analysis did not examine laboratory-related performance indicators, which are Limitationsnecessary to evaluate the quality of results produced by the national serology laboratory.

## 5. Conclusion

In spite of the recent decline in routine immunization coverage and the suboptimal campaign performance, Lesotho has made significant progress in its measles and rubella elimination efforts. Immediately after the COVID-19 pandemic, Lesotho has managed to rapidly recover national immunization services to pre-pandemic performance levels. To sustain the gains made and enhance the quality and coverage of service delivery, several strategic measures should be considered. First, introducing additional second year of life interventions in the routine immunization service could help boost MCV coverage and reduce MCV1-MCV2 dropout rate. Secondly, it is imperative that the denominator issue is addressed through the implementation of periodic vaccination coverage surveys and other robust estimation processes to ensure realistic monitoring of vaccination coverage at all levels. Thirdly, Lesotho needs to maintain good surveillance quality, prioritize documentation of viral genotypes, and leverage the NPEC or establish a dedicated National Verification Committee to guide and document the country’s progress towards formal elimination certification and be submitting annual reports to the African Regional Verification Committee for Measles and Rubella Elimination (AF - RVC). The country should also be conducting periodic measles risk assessments to identify and address programmatic gaps. Finally, future supplemental immunization activities must emphasize robust social mobilization and messaging to ensure all eligible children are reached and information gaps among caregivers are addressed.

What is known about the topic

- Lesotho has been implementing measles mortality reduction strategies since 1996, and has adopted the African Regional measles and rubella elimination goals for 2030.
- Lesotho has historically had relatively high routine immunization coverage and low measles incidence.
- Lesotho was one of the 7 Southern African countries affected by the resurgence of measles, after many years of low incidence, as manifested in the concurrent multi-country measles outbreaks of 2009–2010.

What this study adds:

- Lesotho maintains high MCV1 coverage but will need to work to reduce the drop out rate between MCV1 and MCV2.
- The national level administrative and survey coverage from MR SIAs have been suboptimal, contributing to persistent measles immunity gaps, and explaining the increasing measles and rubella incidence in recent years.
- The country has met the targets for the main measles case-based surveillance performance indicators, but needs to implement detailed case investigation and start documenting the circulating measles and rubella viral strains.
