# Supplementary material for: Progress towards measles and rubella elimination in Lesotho, 2011–2025
Source: PLoS One. 2026 Sep 16;21(9):e0358555. doi: 10.1371/journal.pone.0358555 (PMC13580988; doi:10.1371/journal.pone.0358555)
Supplement: Supplementary file 1 [file pone.0358555.s002.docx]

**Progress Towards Measles and Rubella Elimination in Lesotho**

**Response to editor/reviewers’ comments**

We are very grateful for the review comments provided by the editor and each of the external reviewers of this manuscript. The comments are encouraging and the reviewers appear to share our judgement that Lesotho is close to eliminating measles and rubella. Please see below, in blue, our detailed response to comments.

All page numbers refer to the manuscript file with tracked changes.

**Journal Requirements:**

When submitting your revision, we need you to address these additional requirements.
1. Please ensure that your manuscript meets PLOS ONE's style requirements, including those for file naming.

**Authors’ Response:** We have effected notable changes to align with PLOS ONE's style. These include the removal of the physical address of the corresponding author; listing author affiliations in numbers rather than letters; and changing the font sizes of levels 1 and 2 headings to the recommended 18pt and 16pt fonts respectively.

**Reviewer comments to the Author**

**1. Is the manuscript technically sound, and do the data support the conclusions?**

Reviewer #1: Yes

Reviewer #2: Yes

**Authors’ Response:** Thank you very much for your comment

**2. Has the statistical analysis been performed appropriately and rigorously?**

Reviewer #1: Yes

Reviewer #2: N/A

**Authors’ Response:** Thank you very much for your comment

**3. Have the authors made all data underlying the findings in their manuscript fully available?**

Reviewer #1: Yes

Reviewer #2: Yes

**Authors’ Response:** Thank you very much for your comment

**4. Is the manuscript presented in an intelligible fashion and written in standard English?**

Reviewer #1: Yes

Reviewer #2: Yes

**Authors’ Response:** Thank you very much for your comment

**5. Review Comments to the Author**

**Reviewer #1:**

**1.1. Reviewer comment: Introduction
•**Paragraph 1: please cite the year of reference data related to the information cited on GDP and HIV rates.
•Between paragraph 3 and paragraph 4, I think it would be good to have a couple of bridging sentences to provide some information on the current Regional goals regarding measles and rubella elimination, and the global progress**.**

**Authors’ Response:**

Thank you for your comments on the introduction. We have provided the years for the cited GDP and HIV figures. We have also inserted the required bridging sentences between paragraphs 3 and 4 as recommended.

**1.2. Reviewer comment: Methods**
•The sources of data and methods are clearly indicated.
•Routine immunization: Since we now have the WHO-UNICEF coverage estimates for 2025, I suggest adding the 2025 data.
•Surveillance activities: I suggest moving the first sentence on CRS sentinel surveillance to the end of that paragraph**.**

**Authors’ Response:**

Thank you for your comments on the methods. We have effected the said change by including the recently released WUENIC estimates for 2025 in the analyses. Surveillance activities is in the results section of the manuscript instead. Nonetheless, we have moved the sentence on CRS sentinel surveillance to the end of the said paragraph as you recommended.

**1.3. Reviewer comment: Results**

•Clearly stated and tabulated results.
•Since we now have the WHO-UNICEF coverage estimates for 2025, I suggest adding the 2025 data.

**Authors’ Response:**

Thank you for your comments on the results. We have effected the said change by including the recently released WUENIC estimates for 2025 in the analyses

**1.4. Reviewer comment: Discussion**

This section goes into detail on the interpretation of the data and provides comparison with other countries and other studies.
•Paragraph 4: I suggest the following two small edits
Survey coverage estimates have always been higher than the reported administrative coverage for both routine and supplemental immunization activities in Lesotho, which is suggestive of the existence of a denominator problem inaccuracies. Similar trends were observed in Eritrea with survey results consistently higher than administrative coverage estimates due to inaccurate denominators [17]. It is therefore imperative that the denominator issue is resolved addressed, either through a nationwide census or through scientifically sound methods, in order to obtain credible coverage estimates for both routine and supplemental immunization activities.
•Last Paragraph:
I suggest removing this particular sentence, since the NPEC does not have the role as indicated.
Lesotho relies on the National Polio Eradication Committee (NPEC) to oversee measles and rubella elimination efforts

**Authors’ Response:**

Thank you for your comments on the discussion. We effected the suggested minor edits.

**1.5. Reviewer comment: Limitations**

•The limitations are well stated.

**Authors’ Response:**

Thank you for your feedback.

**1.6. Reviewer comment: Conclusions**
The conclusions are very relevant and emanate from the study findings.
•One possible solution to address the challenge of inaccurate denominators is to implement vaccination coverage surveys. Since censuses are quite outside the scope of immunisation programs, I think it will be good to also recommend periodic coverage surveys as a way of generating better estimates.

**Authors’ Response:**

Thank you for your comment. We effected the suggested minor edits.

**1.7. Reviewer comment: References:**
•Relevant and up to date

**Authors’ Response:**

Thank you for your comment.

**Reviewer #2:**

**2.1. Reviewer comment: General**

Overall, it is an important article and great to highlight national efforts to achieve and maintain elimination. I think that Lesotho could be one of the next countries to be verified measles and rubella elimination. I have included comments below and I think that with a few edits is could be improved.

**Authors’ Response:**

Thank you for your feedback.

**2.2. Reviewer comment: Title**

 Include timeline in the title: "Progress towards Measles and Rubella Elimination in Lesotho, 2025" or "Progress towards Measles and Rubella Elimination in Lesotho, 2011-2025"

**Authors’ Response:**

Thank you for your comment. We have incorporated the timeline in the title.

**2.3. Reviewer comment: Conclusion**

In the conclusion, it would be good to include some discussion on next steps towards measles and rubella elimination - i.e., submitting an annual report to the African Regional Verification Commission for Measles and Rubella Elimination (AF - RVC) or activities toward formalizing the National Verification Committee (NVC). In a quick review of the of the Lesotho measles and rubella data, I think that the country has potentially interrupted endemic transmission of measles and rubella viruses. It would take a more in depth documentation and visualization of the cases based surveillance and immunization data. I think that this study is a good first start and could be a jumping off point to generate additional interested and effort.

**Authors’ Response:**

Thank you for your feedback. Although we already had some next steps outlined in the conclusion, we have added the need for the annual submission of documented progress towards measles-rubella elimination as you recommended.

**2.4. Reviewer comment: Discussion**

It would be good to review the 5 lines of evidence needed for demonstrating, documenting measles and/or rubella elimination in the WER - "Guidance for evaluating progress towards elimination of measles and rubella (it is in the reference #27) and potential outline them in the discussion section.

**Authors’ Response:**

Thank you for your comment. The discussion is already structured along the 5 lines of evidence needed for demonstrating and documenting measles and/or rubella elimination: Population Immunity, Surveillance Performance, Epidemiological Investigation, Molecular Epidemiology and Sustainability and equity. The equity component has just been added before the final sentence of the last paragraph.

**2.5. Reviewer comment: Results**

In table 2, it might be helpful to include the standard for non-measles febrile rash illness rate / 100,000 pop in column 5, ie, 2 and in column 6, include 80% as the standard surveillance goal. It could be in parenthesis.

**Authors’ Response:**

Thank you for your comment. We have made the suggested additions in both columns.

**2.6. Reviewer comment: Results**

In figure 1, it would be helpful to include immunization coverage numbers on the graph and you could add supplementary SIA activities as dots on the graph. You can include more data points to help visual the data.

**Authors’ Response:**

Thank you for your feedback. We have now displayed the immunization coverage numbers on figure 1 as you recommended. SIA coverage figures have been indicated in a separate table.

**2.7. Reviewer comment: Results**
Table 3 could be an epi curve/bar chart with suspected and confirmed case by month or year and could visual help document interruption and elimination of endemic chains.

**Authors’ Response:**

Thank you for your feedback.

**2.8. Reviewer comment: Methods**

Check to make sure that the abbreviations are spelled out prior to use: in the methods section the second sentence needs to have (SIAs) after "supplementary immunization activities" is used.

**Authors’ Response:**

Thank you for your feedback. This is noted. We effected the suggested edit.
