# Supplementary material for: Progress towards measles and rubella elimination in Lesotho, 2011–2025
Source: PLoS One. 2026 Sep 16;21(9):e0358555. doi: 10.1371/journal.pone.0358555 (PMC13580988; doi:10.1371/journal.pone.0358555)
Supplement: Peer review history

# Peer review: Decision Letter 0

**DOI:** 10.1371/journal.pone.0358555.r001

**Authors:** Omar Enzo Santangelo

9 Aug 2026

Dear Dr. Singhateh,

Thank you for submitting your manuscript to PLOS ONE. After careful consideration, we feel that it has merit but does not fully meet PLOS ONE’s publication criteria as it currently stands. Therefore, we invite you to submit a revised version of the manuscript that addresses the points raised during the review process.

==============================

**ACADEMIC EDITOR:**

Dear authors, the manuscript requires minor revisions; please respond to the reviewers' comments point by point.

Kind regards.

For Lab, Study and Registered Report Protocols: These article types are not expected to include results but may include pilot data.

==============================

Please submit your revised manuscript by Sep 08 2026 11:59PM. If you will need more time than this to complete your revisions, please reply to this message or contact the journal office at plosone@plos.org. When you're ready to submit your revision, log on to https://www.editorialmanager.com/pone/ and select the 'Submissions Needing Revision' folder to locate your manuscript file.

- A letter that responds to each point raised by the academic editor and reviewer(s). You should upload this letter as a separate file labeled 'Response to Reviewers'.
- A marked-up copy of your manuscript that highlights changes made to the original version. You should upload this as a separate file labeled 'Revised Manuscript with Track Changes'.
- An unmarked version of your revised paper without tracked changes. You should upload this as a separate file labeled 'Manuscript'.

If you would like to make changes to your financial disclosure, please include your updated statement in your cover letter. Guidelines for resubmitting your figure files are available below the reviewer comments at the end of this letter.

If applicable, we recommend that you deposit your laboratory protocols in protocols.io to enhance the reproducibility of your results. Protocols.io assigns your protocol its own identifier (DOI) so that it can be cited independently in the future. For instructions see: https://journals.plos.org/plosone/s/submission-guidelines#loc-laboratory-protocols. Additionally, PLOS ONE offers an option for publishing peer-reviewed Lab Protocol articles, which describe protocols hosted on protocols.io. Read more information on sharing protocols at https://plos.org/protocols?utm_medium=editorial-email&utm_source=authorletters&utm_campaign=protocols.

As the corresponding author, your ORCID iD is verified in the submission system and will appear in the published article. PLOS supports the use of ORCID, and we encourage all coauthors to register for an ORCID iD and use it as well. Please encourage your coauthors to verify their ORCID iD within the submission system before final acceptance, as unverified ORCID iDs will not appear in the published article. *Only*  the individual author can complete the verification step; PLOS staff *cannot*  verify ORCID iDs on behalf of authors.

We look forward to receiving your revised manuscript.

Kind regards,

Omar Enzo Santangelo

Academic Editor

PLOS One

Journal Requirements:

When submitting your revision, we need you to address these additional requirements.

1. Please ensure that your manuscript meets PLOS ONE's style requirements, including those for file naming. The PLOS ONE style templates can be found at

https://journals.plos.org/plosone/s/file?id=wjVg/PLOSOne_formatting_sample_main_body.pdf and

https://journals.plos.org/plosone/s/file?id=ba62/PLOSOne_formatting_sample_title_authors_affiliations.pdf

2. Please include your tables as part of your main manuscript and remove the individual files. Please note that supplementary tables (should remain/ be uploaded) as separate "supporting information" files

3. If the reviewer comments include a recommendation to cite specific previously published works, please review and evaluate these publications to determine whether they are relevant and should be cited. There is no requirement to cite these works unless the editor has indicated otherwise.

4. Please review your reference list to ensure that it is complete and correct. If you have cited papers that have been retracted, please include the rationale for doing so in the manuscript text, or remove these references and replace them with relevant current references. Any changes to the reference list should be mentioned in the rebuttal letter that accompanies your revised manuscript. If you need to cite a retracted article, indicate the article’s retracted status in the References list and also include a citation and full reference for the retraction notice.

[Note: HTML markup is below. Please do not edit.]

Reviewers' comments:

Reviewer's Responses to Questions

**Comments to the Author**

1. Is the manuscript technically sound, and do the data support the conclusions?

Reviewer #1: Yes

Reviewer #2: Yes

**********

2. Has the statistical analysis been performed appropriately and rigorously?

Reviewer #1: Yes

Reviewer #2: N/A

**********

3. Have the authors made all data underlying the findings in their manuscript fully available?

The PLOS Data policy

Reviewer #1: Yes

Reviewer #2: Yes

**********

4. Is the manuscript presented in an intelligible fashion and written in standard English?

Reviewer #1: Yes

Reviewer #2: Yes

**********

Reviewer #1: This is a detailed review of the measles and rubella elimination status in one specific country – Lesotho. The manuscript is well written, and the language is clear.

Introduction:

•Paragraph 1: please cite the year of reference data related to the information cited on GDP and HIV rates.

•Between paragraph 3 and paragraph 4, I think it would be good to have a couple of bridging sentences to provide some information on the current Regional goals regarding measles and rubella elimination, and the global progress.

Methods:

•The sources of data and methods are clearly indicated.

•Routine immunization: Since we now have the WHO-UNICEF coverage estimates for 2025, I suggest adding the 2025 data.

•Surveillance activities: I suggest moving the first sentence on CRS sentinel surveillance to the end of that paragraph.

Results:

•Clearly stated and tabulated results.

•Since we now have the WHO-UNICEF coverage estimates for 2025, I suggest adding the 2025 data.

Discussion:

This section goes into detail on the interpretation of the data and provides comparison with other countries and other studies.

•Paragraph 4: I suggest the following two small edits

Survey coverage estimates have always been higher than the reported administrative coverage for both routine and supplemental immunization activities in Lesotho, which is suggestive of the existence of a denominator problem inaccuracies. Similar trends were observed in Eritrea with survey results consistently higher than administrative coverage estimates due to inaccurate denominators [17]. It is therefore imperative that the denominator issue is resolved addressed, either through a nationwide census or through scientifically sound methods, in order to obtain credible coverage estimates for both routine and supplemental immunization activities.

•Last Paragraph:

I suggest removing this particular sentence, since the NPEC does not have the role as indicated.

Lesotho relies on the National Polio Eradication Committee (NPEC) to oversee measles and rubella elimination efforts.

•The limitations are well stated.

Conclusions:

The conclusions are very relevant and emanate from the study findings.

•One possible solution to address the challenge of inaccurate denominators is to implement vaccination coverage surveys. Since censuses are quite outside the scope of immunisation programs, I think it will be good to also recommend periodic coverage surveys as a way of generating better estimates.

References:

•Relevant and up to date

Reviewer #2: Overall, it is an important article and great to highlight national efforts to achieve and maintain elimination. I think that Lesotho could be one of the next countries to be verified measles and rubella elimination. I have included comments below and I think that with a few edits is could be improved.

Title: Include timeline in the title: "Progress towards Measles and Rubella Elimination in Lesotho, 2025" or "Progress towards Measles and Rubella Elimination in Lesotho, 2011-2025"

Conclusion: In the conclusion, it would be good to include some discussion on next steps towards measles and rubella elimination - i.e., submitting an annual report to the African Regional Verification Commission for Measles and Rubella Elimination (AF - RVC) or activities toward formalizing the National Verification Committee (NVC). In a quick review of the of the Lesotho measles and rubella data, I think that the country has potentially interrupted endemic transmission of measles and rubella viruses. It would take a more in depth documentation and visualization of the cases based surveillance and immunization data. I think that this study is a good first start and could be a jumping off point to generate additional interested and effort.

WHO country slides has good visualization for Lesotho on slide 95:

https://immunizationdata.who.int/global?topic=Provisional-measles-and-rubella-data&location=

It would be good to review the 5 lines of evidence needed for demonstrating, documenting measles and/or rubella elimination in the WER - "Guidance for evaluating progress towards elimination of measles and rubella (it is in the reference #27) and potential outline them in the discussion section.

In table 2, it might be helpful to include the standard for non-measles febrile rash illness rate / 100,000 pop in column 5, ie, 2 and in column 6, include 80% as the standard surveillance goal. It could be in parenthesis.

In figure 1, it would be helpful to include immunization coverage numbers on the graph and you could add supplementary SIA activities as dots on the graph. You can include more data points to help visual the data.

Table 3 could be an epi curve/bar chart with suspected and confirmed case by month or year and could visual help document interruption and elimination of endemic chains.

Check to make sure that the abbreviations are spelled out prior to use: in the methods section the second sentence needs to have (SIAs) after "supplementary immunization activities" is used.

**********

what does this mean?). If published, this will include your full peer review and any attached files.

If you choose “no”, your identity will remain anonymous but your review may still be made public.

**Do you want your identity to be public for this peer review?**  For information about this choice, including consent withdrawal, please see our Privacy Policy

Reviewer #1: No

Reviewer #2: **Yes:**  Patrick M OConnor

**********

[NOTE: If reviewer comments were submitted as an attachment file, they will be attached to this email and accessible via the submission site. Please log into your account, locate the manuscript record, and check for the action link "View Attachments". If this link does not appear, there are no attachment files.]

To ensure your figures meet our technical requirements, please review our figure guidelines: https://journals.plos.org/plosone/s/figures

You may also use PLOS’s free figure tool, NAAS, to help you prepare publication quality figures: https://journals.plos.org/plosone/s/figures#loc-tools-for-figure-preparation.

NAAS will assess whether your figures meet our technical requirements by comparing each figure against our figure specifications.

# Author comment: Author response to Decision Letter 1

**DOI:** 10.1371/journal.pone.0358555.r002

25 Aug 2026

Progress Towards Measles and Rubella Elimination in Lesotho

Response to editor/reviewers’ comments

We are very grateful for the review comments provided by the editor and each of the external reviewers of this manuscript. The comments are encouraging and the reviewers appear to share our judgement that Lesotho is close to eliminating measles and rubella. Please see below, in blue, our detailed response to comments.

All page numbers refer to the manuscript file with tracked changes.

Journal Requirements:

When submitting your revision, we need you to address these additional requirements.

1. Please ensure that your manuscript meets PLOS ONE's style requirements, including those for file naming.

Authors’ Response: We have effected notable changes to align with PLOS ONE's style. These include the removal of the physical address of the corresponding author; listing author affiliations in numbers rather than letters; and changing the font sizes of levels 1 and 2 headings to the recommended 18pt and 16pt fonts respectively.

Reviewer comments to the Author

1. Is the manuscript technically sound, and do the data support the conclusions?

Reviewer #1: Yes

Reviewer #2: Yes

Authors’ Response: Thank you very much for your comment

2. Has the statistical analysis been performed appropriately and rigorously?

Reviewer #1: Yes

Reviewer #2: N/A

Authors’ Response: Thank you very much for your comment

3. Have the authors made all data underlying the findings in their manuscript fully available?

Reviewer #1: Yes

Reviewer #2: Yes

Authors’ Response: Thank you very much for your comment

4. Is the manuscript presented in an intelligible fashion and written in standard English?

Reviewer #1: Yes

Reviewer #2: Yes

Authors’ Response: Thank you very much for your comment

5. Review Comments to the Author

Reviewer #1:

1.1. Reviewer comment: Introduction

•Paragraph 1: please cite the year of reference data related to the information cited on GDP and HIV rates.

•Between paragraph 3 and paragraph 4, I think it would be good to have a couple of bridging sentences to provide some information on the current Regional goals regarding measles and rubella elimination, and the global progress.

Authors’ Response:

Thank you for your comments on the introduction. We have provided the years for the cited GDP and HIV figures. We have also inserted the required bridging sentences between paragraphs 3 and 4 as recommended.

1.2. Reviewer comment: Methods

•The sources of data and methods are clearly indicated.

•Routine immunization: Since we now have the WHO-UNICEF coverage estimates for 2025, I suggest adding the 2025 data.

•Surveillance activities: I suggest moving the first sentence on CRS sentinel surveillance to the end of that paragraph.

Authors’ Response:

Thank you for your comments on the methods. We have effected the said change by including the recently released WUENIC estimates for 2025 in the analyses. Surveillance activities is in the results section of the manuscript instead. Nonetheless, we have moved the sentence on CRS sentinel surveillance to the end of the said paragraph as you recommended.

1.3. Reviewer comment: Results

•Clearly stated and tabulated results.

•Since we now have the WHO-UNICEF coverage estimates for 2025, I suggest adding the 2025 data.

Authors’ Response:

Thank you for your comments on the results. We have effected the said change by including the recently released WUENIC estimates for 2025 in the analyses

1.4. Reviewer comment: Discussion

This section goes into detail on the interpretation of the data and provides comparison with other countries and other studies.

•Paragraph 4: I suggest the following two small edits

Survey coverage estimates have always been higher than the reported administrative coverage for both routine and supplemental immunization activities in Lesotho, which is suggestive of the existence of a denominator problem inaccuracies. Similar trends were observed in Eritrea with survey results consistently higher than administrative coverage estimates due to inaccurate denominators [17]. It is therefore imperative that the denominator issue is resolved addressed, either through a nationwide census or through scientifically sound methods, in order to obtain credible coverage estimates for both routine and supplemental immunization activities.

•Last Paragraph:

I suggest removing this particular sentence, since the NPEC does not have the role as indicated.

Lesotho relies on the National Polio Eradication Committee (NPEC) to oversee measles and rubella elimination efforts

Authors’ Response:

Thank you for your comments on the discussion. We effected the suggested minor edits.

1.5. Reviewer comment: Limitations

•The limitations are well stated.

Authors’ Response:

Thank you for your feedback.

1.6. Reviewer comment: Conclusions

The conclusions are very relevant and emanate from the study findings.

•One possible solution to address the challenge of inaccurate denominators is to implement vaccination coverage surveys. Since censuses are quite outside the scope of immunisation programs, I think it will be good to also recommend periodic coverage surveys as a way of generating better estimates.

Authors’ Response:

Thank you for your comment. We effected the suggested minor edits.

1.7. Reviewer comment: References:

•Relevant and up to date

Authors’ Response:

Thank you for your comment.

Reviewer #2:

2.1. Reviewer comment: General

Overall, it is an important article and great to highlight national efforts to achieve and maintain elimination. I think that Lesotho could be one of the next countries to be verified measles and rubella elimination. I have included comments below and I think that with a few edits is could be improved.

Authors’ Response:

Thank you for your feedback.

2.2. Reviewer comment: Title

Include timeline in the title: "Progress towards Measles and Rubella Elimination in Lesotho, 2025" or "Progress towards Measles and Rubella Elimination in Lesotho, 2011-2025"

Authors’ Response:

Thank you for your comment. We have incorporated the timeline in the title.

2.3. Reviewer comment: Conclusion

In the conclusion, it would be good to include some discussion on next steps towards measles and rubella elimination - i.e., submitting an annual report to the African Regional Verification Commission for Measles and Rubella Elimination (AF - RVC) or activities toward formalizing the National Verification Committee (NVC). In a quick review of the of the Lesotho measles and rubella data, I think that the country has potentially interrupted endemic transmission of measles and rubella viruses. It would take a more in depth documentation and visualization of the cases based surveillance and immunization data. I think that this study is a good first start and could be a jumping off point to generate additional interested and effort.

Authors’ Response:

Thank you for your feedback. Although we already had some next steps outlined in the conclusion, we have added the need for the annual submission of documented progress towards measles-rubella elimination as you recommended.

2.4. Reviewer comment: Discussion

It would be good to review the 5 lines of evidence needed for demonstrating, documenting measles and/or rubella elimination in the WER - "Guidance for evaluating progress towards elimination of measles and rubella (it is in the reference #27) and potential outline them in the discussion section.

Authors’ Response:

Thank you for your comment. The discussion is already structured along the 5 lines of evidence needed for demonstrating and documenting measles and/or rubella elimination: Population Immunity, Surveillance Performance, Epidemiological Investigation, Molecular Epidemiology and Sustainability and equity. The equity component has just been added before the final sentence of the last paragraph.

2.5. Reviewer comment: Results

In table 2, it might be helpful to include the standard for non-measles febrile rash illness rate / 100,000 pop in column 5, ie, 2 and in column 6, include 80% as the standard surveillance goal. It could be in parenthesis.

Authors’ Response:

Thank you for your comment. We have made the suggested additions in both columns.

2.6. Reviewer comment: Results

In figure 1, it would be helpful to include immunization coverage numbers on the graph and you could add supplementary SIA activities as dots on the graph. You can include more data points to help visual the data.

Authors’ Response:

Thank you for your feedback. We have now displayed the immunization coverage numbers on figure 1 as you recommended. SIA coverage figures have been indicated in a separate table.

2.7. Reviewer comment: Results

Table 3 could be an epi curve/bar chart with suspected and confirmed case by month or year and could visual help document interruption and elimination of endemic chains.

Authors’ Response:

Thank you for your feedback.

2.8. Reviewer comment: Methods

Check to make sure that the abbreviations are spelled out prior to use: in the methods section the second sentence needs to have (SIAs) after "supplementary immunization activities" is used.

Authors’ Response:

Thank you for your feedback. This is noted. We effected the suggested edit.

Attachment Submitted filename: Response to Reviewers.docx

# Peer review: Decision Letter 1

**DOI:** 10.1371/journal.pone.0358555.r003

**Authors:** Omar Enzo Santangelo

2 Sep 2026

Progress TowardsMeasles and Rubella Elimination in Lesotho: 2011-2025

PONE-D-26-35426R1

Dear Dr. Singhateh,

We’re pleased to inform you that your manuscript has been judged scientifically suitable for publication and will be formally accepted for publication once it meets all outstanding technical requirements.

Within one week, you’ll receive an e-mail detailing the required amendments. When these have been addressed, you’ll receive a formal acceptance letter and your manuscript will be scheduled for publication.

An invoice will be generated when your article is formally accepted. Please note, if your institution has a publishing partnership with PLOS and your article meets the relevant criteria, all or part of your publication costs will be covered. Please make sure your user information is up-to-date by logging into Editorial Manager at Editorial Manager® and clicking the ‘Update My Information' link at the top of the page. For questions related to billing, please contact billing support.

If your institution or institutions have a press office, please notify them about your upcoming paper to help maximize its impact. If they’ll be preparing press materials, please inform our press team as soon as possible -- no later than 48 hours after receiving the formal acceptance. Your manuscript will remain under strict press embargo until 2 pm Eastern Time on the date of publication. For more information, please contact onepress@plos.org.

Kind regards,

Omar Enzo Santangelo

Academic Editor

PLOS One

Additional Editor Comments (optional):

Reviewers' comments:

Reviewer's Responses to Questions

**Comments to the Author**

Reviewer #1: All comments have been addressed

Reviewer #2: All comments have been addressed

**********

2. Is the manuscript technically sound, and do the data support the conclusions?

Reviewer #1: Yes

Reviewer #2: Yes

**********

3. Has the statistical analysis been performed appropriately and rigorously?

Reviewer #1: Yes

Reviewer #2: N/A

**********

4. Have the authors made all data underlying the findings in their manuscript fully available?

The PLOS Data policy

Reviewer #1: Yes

Reviewer #2: Yes

**********

5. Is the manuscript presented in an intelligible fashion and written in standard English?

Reviewer #1: Yes

Reviewer #2: Yes

**********

Reviewer #1: The authors have accommodated all the comments provided by the reviewers. The current version of the manuscript clarifies all the points raised, and does not have any methodological or any editorial errors to address. Thank you

Reviewer #2: Manuscript looks great and I really appreciate the efforts of the authors in putting together all of the data and making the recommended corrects. Just one additional thought for the discussion for your consideration - in the second to last paragraph - some countries has used their NCC for polio eradication as way to rapidly set up their NVC for measles and rubella elimination. And in the conclusion - I would suggest changing "maintain good surveillance quality" to "maintain high quality, laboratory supported surveillance". I look forward to Lesotho being among the countries in the African Region that are verified for measles and rubella elimination.

**********

what does this mean?). If published, this will include your full peer review and any attached files.

If you choose “no”, your identity will remain anonymous but your review may still be made public.

**Do you want your identity to be public for this peer review?**  For information about this choice, including consent withdrawal, please see our Privacy Policy

Reviewer #1: No

Reviewer #2: **Yes:** Dr Patrick M O'Connor

**********

# Editor report: Acceptance letter

**DOI:** 10.1371/journal.pone.0358555.r004

**Authors:** Omar Enzo Santangelo

PONE-D-26-35426R1

PLOS One

Dear Dr. Singhateh,

I'm pleased to inform you that your manuscript has been deemed suitable for publication in PLOS One. Congratulations! Your manuscript is now being handed over to our production team.

At this stage, our production department will prepare your paper for publication. This includes ensuring the following:

* All references, tables, and figures are properly cited

* All relevant supporting information is included in the manuscript submission,

* There are no issues that prevent the paper from being properly typeset

You will receive further instructions from the production team, including instructions on how to review your proof when it is ready. Please keep in mind that we are working through a large volume of accepted articles, so please give us a few days to review your paper and let you know the next and final steps.

Lastly, if your institution or institutions have a press office, please let them know about your upcoming paper now to help maximize its impact. If they'll be preparing press materials, please inform our press team within the next 48 hours. Your manuscript will remain under strict press embargo until 2 pm Eastern Time on the date of publication. For more information, please contact onepress@plos.org.

You will receive an invoice from PLOS for your publication fee after your manuscript has reached the completed accept phase. If you receive an email requesting payment before acceptance or for any other service, this may be a phishing scheme. Learn how to identify phishing emails and protect your accounts at https://explore.plos.org/phishing.

If we can help with anything else, please email us at customercare@plos.org.

Thank you for submitting your work to PLOS One and supporting open access.

Kind regards,

PLOS One Editorial Office Staff

on behalf of

Dr. Omar Enzo Santangelo

Academic Editor

PLOS One
